# Dog and cat exposures to drugs of abuse identified by the California animal health and food safety laboratory system 2013–2023

**DOI:** 10.3389/fvets.2024.1372614

**Published:** 2024-03-07

**Authors:** Sigal Klainbart, Chelsea A. Sykes, Robert H. Poppenga

**Affiliations:** ^1^California Animal Health and Food Safety Laboratory System, University of California, Davis, Davis, CA, United States; ^2^Department of Small Animal Emergency and Critical Care, The Veterinary Teaching Hospital, Koret School of Veterinary Medicine, The Robert H. Smith Faculty of Agricultural, Food & Environment, The Hebrew University of Jerusalem, Rehovot, Israel

**Keywords:** illicit, recreational, amphetamine, methamphetamine, opiates, liquid chromatography-mass spectrometry, toxicology

## Abstract

**Introduction:**

While known animal exposures to human “drugs of abuse” (DA) were previously considered relatively uncommon in veterinary medicine, the trends are changing. Marijuana and amphetamines are among the 20 toxicants most frequently consulted about with the Pet Poison Helpline. When such exposures occur, they are typically considered emergencies.

**Methods:**

This retrospective study describes confirmed cases of DA exposure in pets from the California Animal Health and Food Safety Laboratory System (CAHFS), 2013–2023.

**Results:**

Fifty-seven samples tested positive for DA through liquid chromatography with tandem mass spectrometry analysis (qualitative method). In 75% (43/57) of the DA screen tests, the detected drugs included amphetamine-type stimulants and metabolites (methamphetamine, amphetamine, or both). In 47% (27/57) of cases, a combination of more than one drug group was found. Most cases were diagnosed from a urine specimen. In at least 32% (18/57) of cases, the samples were submitted due to suspicions of animal cruelty, and at least 41% (23/57) of the patients were deceased when the samples were submitted.

**Discussion:**

More studies on the prevalence of illicit drugs in small animals, using confirmatory testing, are warranted to fully understand the significance of this emerging toxicological hazard in veterinary medicine.

## 1 Introduction

Drug use disorders carry a lifetime prevalence of about 10% in the general American population, representing more than 23 million adults who are struggling with problematic drug use (1). Despite the well-known deleterious consequences of addiction on physical health, psychology, and quality of life, only a small fraction of people with alcohol or drug use disorders receive any treatment (2). While known animal exposures to human “drugs of abuse” (DA) were considered relatively uncommon in veterinary medicine, the trends are changing, and marijuana and amphetamines are among the 20 toxicants most frequently consulted about with the Pet Poison Helpline (3). When such exposures occur, they are typically considered emergencies. Given the increasing illicit drug use and the addictive nature of many of these compounds in humans, most of these substances are subject to stringent regulation (4). Owners often hesitate to acknowledge the possibility of their pets being exposed to illicit drugs until the animals are in severe distress. Veterinarians need to be well-versed in the most frequently encountered drugs of abuse, the potential clinical courses these exposures can take, and appropriate therapeutic approaches. Additionally, the majority of “street” drugs are not pure and may consist of combinations of substances, making the clinical assessment more complex (5, 6).

The purpose of the present retrospective study was to describe confirmed cases of DA exposure in pets from the *California Animal Health and Food Safety Laboratory System* (CAHFS).

## 2 Materials and methods

The Toxicology Section of CAHFS conducts a qualitative “drugs-of-abuse screen test” by Liquid Chromatography with tandem mass spectrometry (LC–MS/MS) (Table 1). LC–MS/MS is an advanced analytical method that merges the separation capabilities of liquid chromatography with the exceptional sensitivity and specificity of mass analysis offered by triple quadrupole mass spectrometry. The analytical method is qualitative. When analyzing urine samples: a 1-gram sample of urine is incubated with beta-glucuronidase for 2 h at 65°C, and then diluted with Phosphate-buffered saline (PBS). It is purified further using solid phase extraction, and following this blown down to dryness and brought back up in methanol. Analysis is then done by LC–MS/MS with any positive identifications determined by comparison of mass spectra to reference standards. When using tissue samples (e.g., liver, kidney): a 2-gram sample of tissue is combined with PBS and homogenized. Two aliquots are taken for additional extraction, first incubating with beta-glucuronidase for 2 h at 65°C. One aliquot is then adjusted to pH >10 and extracted with methylene chloride while the second is adjusted to pH < 2 and extracted with hexane:ethyl acetate. Following centrifugation, extracts are blown down to dryness and brought back up in methanol. Analysis is then done by LC–MS/MS with any positive identifications determined by comparison of mass spectra to reference standards. Specimens arrive at the lab from across the USA, sent by veterinarians, concerned owners, animal cruelty prevention agencies, law enforcement agencies, and pathology labs. It is not an emergency service; results are typically reported within 10–14 business days. For each received sample, data was collected, to the best extent possible, from the submission form or through a phone call. Available data typically includes information about the exposed species, gender, age, specimen collection location, a brief medical history, suspected or observed exposure, suspected or observed route of exposure, observed clinical effects, and any treatments administered. A total of 175 cases were submitted for drugs-of-abuse screening from January 1st, 2013 to September 18th, 2023.

**Table 1:** Drugs of abuse screen by Liquid Chromatography with tandem mass spectrometry (LC-MS/MS) at the Toxicology Section of the *California Animal Health & Food Safety Laboratory—*University of California—Davis (CAHFS) and corresponding reporting limits.

Analyte	Reporting limit	Units
Cocaine	50	ppb
Benzoylecgonine (BGE)	100	ppb
Norcocaine	50	ppb
Ephedrine	100	ppb
Lysergic acid diethylamide (LSD)	50	ppb
Methamphetamine	50	ppb
3,4-methylenedioxy-methamphetamine (MDMA)	50	ppb
Nicotine	100	ppb
Phentermine	100	ppb
Psilocin	150	ppb
Delta9-Tetrahydrocannabinol (THC)	50	ppb
THC-OH	100	ppb
Methadone	50	ppb
Morphine	50	ppb
THC-COOH	100	ppb
1-Pentyl-3-(1-naphthoyl) indole (JWH-018)	50	ppb
1,1-Dimethylheptyl- 11-hydroxy- tetrahydrocannabinol (HU-210)	50	ppb
Fentanyl	50	ppb
Norfentanyl	50	ppb
Heroin	50	ppb
6-monoacetylmorphine	50	ppb
Midazolam	50	ppb
Alpha-OH-midazolam	50	ppb
1-butyl-3-(1-naphthoyl) indole (JWH-073)	50	ppb

ppb, parts per billion.

## 3 Results

**Figure 1:**
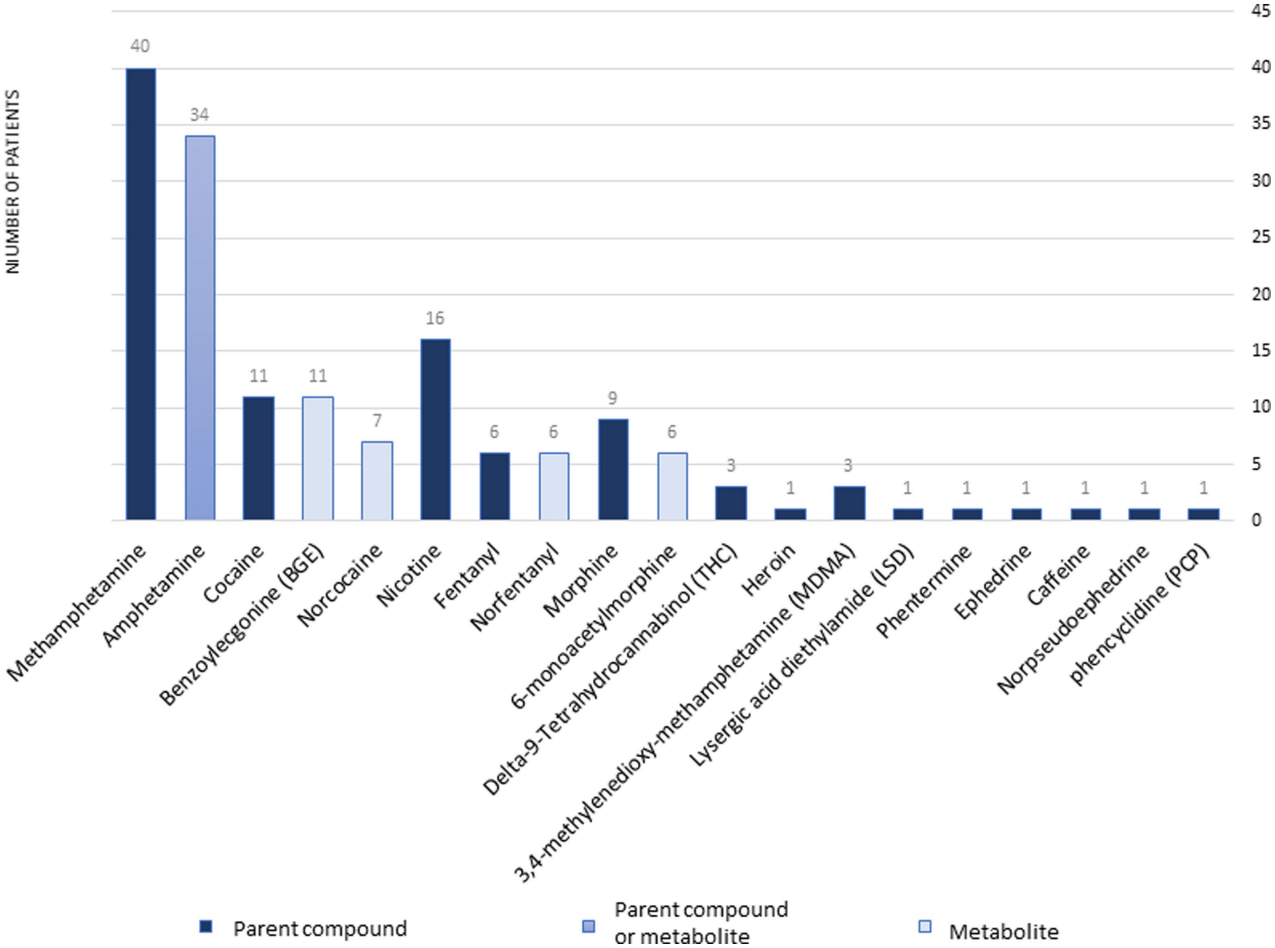
The types and distribution of drugs of abuse diagnoses at the Toxicology Section of the *California Animal Health & Food Safety Laboratory*—University of California—Davis (CAHFS) between January 1, 2013 and September 18, 2023.

**Figure 2:**
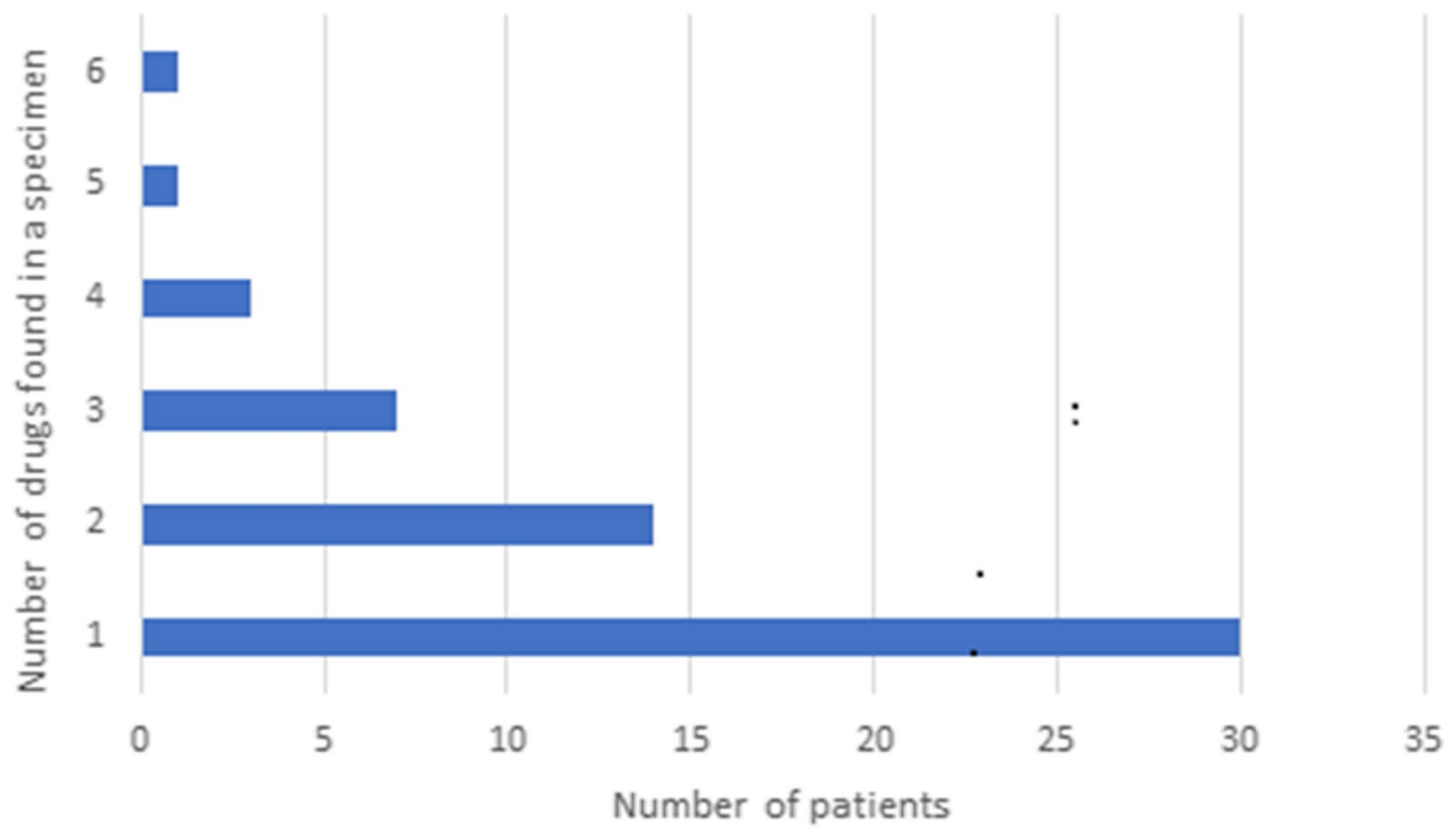
Distribution of multiple drugs of abuse in samples presented to the Toxicology Section of the *California Animal Health & Food Safety Laboratory*—University of California—Davis (CAHFS) between January 1, 2013 and September 18, 2023.

**Figure 3:**
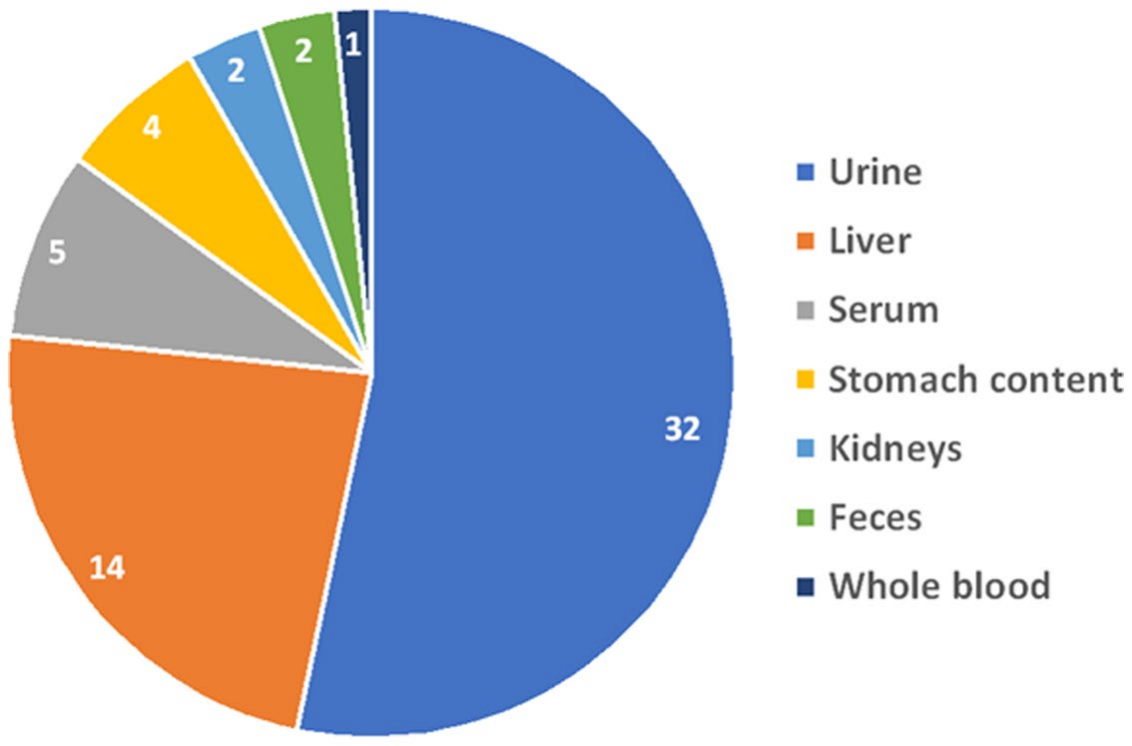
Distribution of the specimens that were evaluated for drugs of abuse at the Toxicology Section of the *California Animal Health & Food Safety Laboratory*—University of California—Davis (CAHFS) between January 1, 2013 and September 18, 2023.

Fifty-seven samples tested positive for DA: 53 were from dogs, and 4 were from cats. Among the dogs, 26 were of unknown breed, 4 were Miniature Pinschers, 4 were Pomeranians, 4 were Chihuahuas, 3 were German Shepherds, 2 were Yorkshire Terriers, 2 were Bull Terriers, and there was 1 each of the following breeds: Toy poodle, Shar-Pei, Alaskan Klee Kai, Terrier mix, Shih Tzu, Collie, Mixed breed, and Belgian Malinois. All 4 cats were of unknown breed. Gender was unknown for 8 patients, there were 17 males, 9 neutered males, 19 females, and 4 spayed females. The median age was 36 months (range, 4–180 months). The types and distribution of DA detected are summarized in Figure 1. In 27 cases (47%), multiple drugs were found in the sample (Figure 2). We did not consider cases in which metabolites of a drug were diagnosed alongside the “parent drug” as “multiple drugs”; e.g., there were 34 cases in which methamphetamine and amphetamine were detected together, 7 cases in which cocaine, benzoylecgonine (BGE), and norcocaine were detected together, and 4 more cases where cocaine and BGE were detected together, in 6 cases fentanyl and norfentanyl were detected together, and in 6 cases morphine and 6-monoacetylmorphine were detected together. The specimens evaluated for DA are summarized in Figure 3, in only 4 cases was more than 1 specimen tested: liver and stomach content in 2 cases, and liver and kidney, and urine and stomach content in 2 others. When compared, in one case, liver and kidney specimens were both positive for amphetamine and methamphetamine, in the second case, liver and stomach content were both positive for amphetamine and methamphetamine, while the stomach content was also positive for THC, heroin, and 6-monoacetylmorphine; in the third case, liver and stomach content were both positive for amphetamine, methamphetamine, and nicotine, while the stomach content was also positive for LSD; in the fourth case, urine sample and stomach content were positive for BGE, while stomach content was also positive for cocaine.

In 20 cases (35%), the history was consistent with highly suspected or known exposure to drugs of abuse. In 20 cases (35%), the reason for the DA screen test was not given. In 19 cases (33%), the samples were sent by the treating veterinarian as part of the medical workup, and in 18 cases (32%), the samples were sent as part of a legal or cruelty investigation. For 18 cases (32%) the outcome was unknown, 14 patients (24%) were alive, and 25 (44%) were dead when samples were submitted.

Table 2 summarizes the known clinical history and reported clinical signs of 40 out of 57 patients whose samples were submitted to the CAHFS for DA screening. Blood test results, (complete blood count and serum biochemistry results) are provided as general comments and not exact values and were available for only five patients, all of whom were dogs. Four of these patients experienced methamphetamine intoxication, while one suffered from 3,4-methylenedioxy-methamphetamine (MDMA) intoxication. All five patients exhibited elevated liver enzymes, three presented with azotemia, two with hypoglycemia, two with hypokalemia, one with mild hyperglycemia, and one with mildly elevated bilirubin concentration.

**Table 2:** Summary of the known clinical history and reported clinical signs of 40 out of 57 patients whose samples were submitted to the CAHFS laboratory for drugs of abuse screening.

Case #	Species	Breed	Clinical history	Reported results of clinical exam	Drugs diagnosed	Outcome
1	Dog	Toy poodle	Known fentanyl and methamphetamine exposure. Has happened several times previously	Unknown	Methamphetamine + amphetamine	Alive
2	Dog	German shepherd	Unknown	Hyperthermia (>110°F), respiratory distress, rigid musculature. Arrested	Methamphetamine + amphetamine	Dead
3	Dog	Unknown	Duration of Illness: one day. May have ingested methamphetamine	Recumbency, tachycardia, respiratory distress. Arrested	Methamphetamine + amphetamine	Dead
4	Dog	Miniature pinscher	Duration of Illness: one day. Concern for Methamphetamine ingestion	Laterally recumbency, obtundation, thrashing and vocalizing, mydriasis OU	Methamphetamine	Dead
5	Dog	Unknown	Found deceased after a domestic dispute	Unknown	Fentanyl	Dead
6	Cat	Unknown	the owner found the kitten in the roommate’s room—the roommate was on drugs.	Distress, panting heavily, dilated pupils, shaking, treated with supportive care, was sent home with cyproheptadine and died a few hrs. later	Methamphetamine + amphetamine	Dead
7	Dog	German shepherd	Owner admitted to giving methamphetamine to his dog, commented that he was not sure if he administered intra-rectally or vaginally.	Autopsy: the reproduction tract was grossly normal. The colon contained soft feces and foreign material, food packaging, but no material that resembled drugs	Methamphetamine + amphetamine	Dead
8	Dog	Bull terrier	Found dead in a car with drug paraphernalia, heroin, methamphetamine	Unknown	Methamphetamine + amphetamine + morphine + 6-monoacetylmprphine	Dead
9	Dog	Unknown	Documented exposure to Fentanyl and Naloxone. Positive on screening* for multiple drugs (urine)	No clinical signs of intoxication	Methamphetamine + amphetamine + norcocaine + nicotine + norfentanyl	Alive
10	Cat	Unknown	Positive on screening* for multiple drugs (urine)	No clinical signs of intoxication	Methamphetamine + amphetamine + cocaine + benzoylecgonine (BGE) + norcocaine	Alive
11	Dog	Unknown	Duration of Illness: two days. Presented for acute altered State. Owner reported that the patient may have ingested illegal drugs (unsure which type)	Ataxia/hypermetria, mydriasis OD	Amphetamine + nicotine + norfentanyl	Alive
12	Dog	Unknown	Possible exposure to various drugs of abuse per owner	Unknown	Methamphetamine + amphetamine + nicotine	Dead
13	Dog	Unknown	The dog was found deceased in a car. The dog was in the car for an unknown length of time. The owner was under the influence of unknown drugs at the time of the dog’s death	Unknown	Methamphetamine + nicotine	Dead
14	Dog	Unknown	Duration of Illness: three days. Presented with a history of seizure and neurologic signs. Possibly ingested paroxetine and/or thyroxine. Positive on screening* for amphetamine, MDMA, methamphetamine, and benzodiazepine (urine) (was given diazepam upon initial presentation)	Temperature of 109.3, tachypnea, tachycardia, dull mentation, tremors	3,4-methylenedioxy-methamphetamine (MDMA) + alpha-Hydeoxymidazolam	Unknown
15	Dog	Unknown	Exposed to methamphetamine, also possibly THC	Lethargy, seizures, vomiting. Arrested	Methamphetamine + amphetamine + morphine + 6-monoacetylmprphine	Dead
16	Dog	Yorkshire terrier	Duration of Illness: three days. Was found vocalizing, salivating, and unable to walk, after being left alone in son’s room	Lateral recumbency, with abnormal mentation, foaming at the mouth, weak limbs neurological reflexes, CP deficits, tremors of the head and neck, tachycardia, hypothermia	Amphetamine + cocaine + nicotine	Alive
17	Dog	Unknown	Owner called and said the dog got into fentanyl powder, treated by fluids and Naloxone	Unknown	Fentanyl + Norfentanyl	Alive
18	Dog	Unknown	Single seizure-like episode	Compulsive circling, ataxia (mild, generalized), reduced proprioception × 4, hyperactive behavior (of at least one-week duration). MRI= Several chronic cerebral micro bleeds. CSF analysis showed no abnormalities	Cocaine + benzoylecgonine (BGE)	Unknown
19	Cat	Unknown	Possible exposure to methamphetamine	Unknown	Methamphetamine + amphetamine + benzoylecgonine (BGE)	Unknown
20	Dog	Unknown	Acute onset of lethargy and disorientation, suspect THC despite negative results on in-house test	Unknown	Cocaine + benzoylecgonine (BGE) + norcocaine + nicotine	Unknown
21	Dog	Unknown	Duration of illness 3 weeks. Blunt force trauma over several weeks and methamphetamine administration	Necropsy: subdural hematuria, multiple broken ribs and liver lacerations with hemoabdomen	Methamphetamine + amphetamine	Dead
22	Dog	Pomeranian	Dog is suspected of being killed by the owner through force-feeding it pills and then smothering it	Unknown	Methamphetamine + amphetamine	Dead
23	Dog	Unknown	Duration of Illness: 2 h. Three dogs in the household with acute onset intractable seizures.	Seizures. Not responsive to treatment with diazepam and propofol. Euthanized.	Cocaine + benzoylecgonine (BGE) + nicotine	Dead
24	Dog	Shar-pei	Three dogs seized for suspicion of drug exposure. Rapid screen positive for cocaine, methamphetamine, amphetamine, and opioids. Negative for THC. Likely exposure to Fentanyl.	Unknown	Amphetamine + Cocaine + benzoylecgonine (BGE) + fentanyl + morphine + nicotine	Unknown
25	Dog	Unknown	Patient presented for agitation, hyperactivity. Amphetamines in the home	Agitation, hyperactivity	Methamphetamine + amphetamine	Unknown
26	Cat	Unknown	Acute onset mydriasis, disorientation, agitation, ataxia; gradual resolution of signs over approximately 5 days. The owner takes phentermine at home but no known access	MRI normal.	Phentermine	Alive
27	Dog	American Pit Bull Terrier	Unknown	Sever ataxia, circling, vomiting, anisocoria and mydriasis OD	Amphetamine + Cocaine + benzoylecgonine (BGE) + norcocaine + nicotine + ephedrine + norfentanyl + morphine	Unknown
28	Dog	Miniature pinscher	Dog ate Heroin, transported to a veterinary clinic, died at clinic	Unknown	Methamphetamine + amphetamine + morphine	Dead
29	Dog	Pomeranian	Presented with another dog (2-month-old puppy who developed seizures that lasted 45 min and then arrested). Owner had heard the two dogs barking in yard and found both circling and frantic. 1.5 h later the puppy started seizing	Extremely agitated/frantic, circling, symmetrically dilated pupils, temperature −103.8	Methamphetamine + amphetamine	Unknown
30	Dog	German shepherd	Potential adverse drug reaction after administration of Trifexis. died shortly afterwards	Autopsy: no striking gross pathology	Methamphetamine + amphetamine	Dead
31	Dog	Terrier mix	Acute onset circling, the owner took dog for a walk on leash. Acutely became restless, excited, staggering around, and circling. Would not eat or drink.	Circling in either direction. No postural deficits, no CN deficits except mydriasis OU, decreased and incomplete PLR OU and absent menace OD. Temperature on presentation was 104°F. The dog was leaking clear urine at presentation, 3 hours later, he had pigmenturia of dark brown color	Methamphetamine + amphetamine	Alive
32	Dog	Unknown	Possible illegal drug overdose-	Malignant hyperthermia and hyperactivity	Methamphetamine + amphetamine + nicotine	Dead
33	Dog	Unknown	Marijuana is present in house but owner believes patient has no access	Episode of acute hyperactivity followed by prolonged (>12 h) of ataxia and hyperesthesia, urinary incontinence and mydriasis	Morphine	Unknown
34	Dog	Chihuahua	Acting strange. Owner's brother has been watching the dog and noted strange behavior. Toxin/foreign material exposure is unknown due to many people living in household.	Distended abdomen and abnormal mentation	Methamphetamine	Alive
35	Dog	Shih Tzu	The day before presentation the dog seemed subdued, on the day of presentation swinging her head from side to side, the owners occasionally give her coffee, which she drinks readily. Additionally, one of the owner’s smokes, and the dog has been known to investigate the ash trays	Abnormal head movements and increased activity	Methamphetamine + amphetamine + caffeine	Alive
36	Dog	Chihuahua	The owner reports Chunk was seen eating something at the park. Since that time became unresponsive to the owners and developed muscle fasciculations.	Behavior change and tremors.	Amphetamine + THC	Alive
37	Dog	Collie	Excessive pacing and lack of owner recognition. had been running back and forth. No known exposure to toxins, human medication or drugs of abuse	Excessive pacing and lack of owner recognition	Methamphetamine + amphetamine + MDMA + norpseudoephedrine	Alive
38	Dog	Mixed breed	Acute onset seizures. found in a ditch in the backyard trembling. The night prior to presentation he was at a friend's house. Vomited after eating grass at the friend's house. There are numerous construction projects at the friend's house	Convulsions every 1–2 min. Between convulsions—dully responsive. During seizure becomes stiff, ventral flexes neck and rolls over onto back with feet straight up in the air. There is fast nystagmus and ventral strabismus. Temperature 103.2, tachycardia, tachypnea	Methamphetamine	Alive
39	Dog	Belgian Malinois	Police drug detection dog	Confused and wobbling	THC + MDMA + phencyclidine (PCP)	Alive
40	Dog	Unknown	Was left with an individual for about 2 h. The owners were notified that the dog died suddenly. Suspected exposure to fentanyl, methamphetamine, and/or crack cocaine.	Unknown	Methamphetamine + amphetamine + norcocaine + fentanyl	Dead

CAHF, California Animal Health and Food Safety Laboratory, University of California – Davis. *Screening, bedside point of care toxicology screens.

In seven cases, a point-of-care (POC) test was conducted before hospital admission. A POC test is a quick and convenient medical diagnostic tool that detects drugs or their metabolites in biological samples (e.g., urine, saliva, blood, or sweat). It uses a specialized kit with reagents or antibodies to identify specific substances, producing visible results within minutes. In two cases, the available information indicated the presence of “multiple drugs.” These cases involved a dog and a cat, where the DA screen revealed the presence of methamphetamine, amphetamine, norcocaine, nicotine, and norfentanyl in the dog sample, and methamphetamine, amphetamine, cocaine, benzoylecgonine (BGE), and norcocaine in the cat sample. In another case, the POC test detected amphetamine, MDMA, methamphetamine, and benzodiazepines, while LC–MS–MS confirmed MDMA and α-hydroxymidazolam. Yet another dog was diagnosed with cocaine, methamphetamine, amphetamine, and opiate exposure, with LC–MS/MS detecting amphetamine, cocaine, BGE, fentanyl, morphine, and nicotine. In 2 cases POC was positive for methadone, which was not detected by LC–MS/MS, but was positive for doxylamine a known cross-reacting OTC drug. In the last case, it was only mentioned that the kit was negative for THC, but LC–MS/MS identified the presence of cocaine, BGE, norcocaine, and nicotine.

## 4 Discussion

Parallel to the increasing trend in illicit drug use among humans, and in many cases multiple DA use, the intoxication of pets with recreational or illicit substances has become increasingly prevalent in the past decade. Such exposure can occur accidentally, intentionally, or through malicious means. Furthermore, dogs, known for their wandering tendencies and indiscriminate eating habits, are particularly vulnerable to various forms of poisoning, including that caused by illicit drugs (4). Other important exposure scenarios include active duty or training of drug detection dogs (7). and the use of animals, especially dogs, as “pack mules” for the illegal transport of drugs. In the latter scenario, dogs are either fed baggies filled with the drug or baggies are surgically implanted in the peritoneum. Death may occur from either the drug itself (leakage of drug from its container) or secondary to infection following a non-sterile surgical technique (8).

In the present study, in at least 32% of cases, the samples were submitted to CAHFS due to suspicion of cruelty to animals. In 75% of cases, the detected drug combinations included methamphetamine, amphetamine, or both. Methamphetamine, amphetamine, methylenedioxy-methamphetamine, and other designer amphetamines collectively belong to the group of drugs known as amphetamine-type stimulants (ATS) (9). Amphetamines are part of a class of psychotropic drugs initially developed for human use in the treatment of conditions such as attention deficit hyperactivity disorder (ADHD) and narcolepsy. Amphetamine is classified as α-methylphenethylamine (10). In contrast to some AST’s (e.g., amphetamine and methylphenidate) which have recognized clinical applications and advantages, methamphetamine and methamphetamine designer drugs are potent psychostimulants known for their high addiction potential (9, 11). Methamphetamine currently ranks as the second most widely abused drug worldwide and ATS have become the most popular illegal psychostimulants in the world (9, 11). Experiments investigating the disposition and fate of amphetamine and methamphetamine in the body confirm that approximately 30–40% of the ingested dose is excreted unchanged in the urine (12, 13). Other studies indicate that about 5–7% of methamphetamine undergoes N-demethylation to amphetamine as the primary metabolite, while amphetamine is not metabolized into methamphetamine (13–15). Consequently, when pure methamphetamine is consumed, the concentration ratio of methamphetamine to amphetamine should be greater than one (15). This may explain the reason for diagnosing both methamphetamine and amphetamine in most (85%) of the examinations verifying exposure to methamphetamine inhalation, although the CAHFS DA tests are qualitative rather than quantitative. ATS are quickly absorbed through the gastrointestinal tract, although for prescription ATS their absorption is delayed when using sustained-release products (SRP). The oral bioavailability of methamphetamine is estimated to be 67% with rapid, widespread distribution throughout most body tissues (16). Amphetamines may also be absorbed in dogs through inhalation or contact with mucous membranes (4, 8). Peak plasma concentrations of amphetamine are typically reached within 1–3 h following ingestion, except when a SRP has been taken. Amphetamine is highly lipophilic, allowing it to readily penetrate the blood–brain barrier (4, 8). In humans, approximately 70% of an oral methamphetamine dose is eliminated through urine within 24 h, with the parent drug accounting for 30–50% and the amphetamine metabolite contributing about 10%. Given that urine serves as the primary route for the elimination of methamphetamine and its primary metabolite, amphetamine, it is considered a suitable specimen for toxicological analysis, as well as serum/plasma. Unfortunately, urine may not be accessible during necropsy (11, 17, 18). Other samples that can be sent for LC–MS/MS analysis in dogs may include liver, kidney tissues, and stomach contents, which often have higher drug concentrations compared to other tissues. These samples may be especially useful for postmortem confirmation (11). Amphetamine is eliminated in dogs within approximately 6 h when the average urine pH is around 7.5, and within 3.3 h when the average urine pH is around 6.6 (8).

Given the abundant use of ATS as an illicit drug in humans in the US, there is no surprise in the finding that it was also the most common to be found among the pet samples submitted to CAHFS. The most common clinical signs reported for methamphetamine/amphetamine intoxication in the present study included seizures, agitation, hyperreactivity, tremors, ataxia, circling, mydriasis, tachypnea, tachycardia, and hyperthermia, all consistent with sympathomimetic effects. Forty-three percent of patients who tested positive for methamphetamine/amphetamine were dead when samples were submitted. Nevertheless, while considering these findings, it is important to remember that many of the patients were exposed to more than one drug. The prognosis for animals with ATS intoxication depends on the consumed dose, the time elapsed between exposure and presentation, and the severity of clinical signs, and is overall considered fair, but there is an information gap in formulating a prognosis (8). Differential diagnosis for ATS intoxication may include exposure to other drugs such as cocaine, ephedrine, pseudoephedrine, methylxanthines, caffeine, selective serotonin reuptake inhibitors (SSRIs), tremorgenic mycotoxins, metaldehyde, or strychnine, which may cause similar central nervous system and/or cardiovascular stimulation, as well as conditions like pheochromocytoma.

Specific treatments were rarely mentioned in the provided case histories. In general, the ideal approach should focus on prevention and/or controlling life-threatening central nervous system and cardiovascular signs. This may include decontamination methods, such as emesis, gastric lavage (depending on the timing and amount ingested), activated charcoal, hospitalization for intravenous fluid treatment, and addressing symptomatic signs. These treatments could involve the use of phenothiazines for agitation, cyproheptadine as a serotonin antagonist, beta-blockers for managing tachycardia and hypertension, and other antiarrhythmics (e.g., lidocaine, procainamide) for severe arrhythmias. Methocarbamol can be employed for tremors, barbiturates for seizures, and the use of propofol or inhaled anesthesia in cases of uncontrolled seizures. The use of diazepam is controversial in treating pets with ATS intoxication, as it may increase dysphoria, paradoxical stimulation, and morbidity. Unfortunately, there is no specific antidote available for ATS intoxication, so monitoring and prompt treatment for signs of hyperthermia, cardiac arrhythmias (including ECG monitoring), and blood pressure are crucial (4, 8, 19). The use of IV lipid emulsion (ILE) infusion has been mentioned in cases of ATS intoxication (20–22). However, its efficacy has not been definitively approved, and some authors state that ILE is not indicated for cases of amphetamine toxicosis, due to a lack of known efficacy and the potential for clinical signs to worsen with the administration because it can decrease the efficacy of some therapeutic medications (23).

Interestingly, despite the staggering increase in opioid-related morbidity and mortality among humans in the US that has earned the name “the opioid epidemic” (24), and that the prevalence of cocaine use has only increased modestly, cocaine-involved overdose mortality has risen dramatically (25). Opioids (16%), synthetic opioids (14%), and cocaine (21%) were presented in relatively small numbers in the present study, often in combination with other drugs, especially ATS.

Only 7 out of 175 specimens tested positive solely for Delta-9-Tetrahydrocannabinol (THC). These cases were not included in the study’s results. All in all, with current trends in cannabinoid legalization and reduced or eliminated state penalties for cannabis possession, the odds of a cannabis poisoning call to poison control centers have increased (26). Despite this fact, it appears that testing at CAHFS for THC as a recreational drug “intoxication” is less common. This is likely due to the fact that clinicians are more aware of the possibility of marijuana exposure, are familiar with the clinical signs of marijuana exposure, utilize the cost-effective urine drug screening test (UDST), and recognize that marijuana intoxication is a relatively mild clinical condition that resolves quickly with mostly no adverse effects (27). In 3 out of 57 cases in the present study, THC was found in conjunction with other drugs of abuse and was therefore reported.

Nicotine was found in 29% of samples, mostly (63% of nicotine exposures) as a trace exposure in the sample, and always in combination with other drugs. These exposures were probably not a “true” nicotine intoxication, but rather an indication of “passive smoking.” This finding would not be unexpected in a pet living in an environment that may contain illicit or recreational drugs (28). The significance of a “trace exposure in the sample” can be explained by the fact that LC–MS/MS was not designed as a quantitative test since detected concentrations do not contribute to the interpretation. The test is designed to determine exposure. A sample is spiked with a specific analyte (e.g., nicotine) concentration that is as low as possible but still readily detected. This is considered the “reporting limit.” If a signal is higher than the reporting limit for that analyte, the result is reported as “positive.” If a positive signal for an analyte is detected below the reporting limit but sufficiently above the analytical “background” signal (3–5 times the background signal), it is reported as a “trace.” If a signal is not detected or not detected sufficiently above the background signal, the result is considered “negative.”

In the present study, only 7 cases reported the use of point-of-care (POC) on-site urine multidrug tests or UDST. These cases seem to exhibit a reasonable correlation with the LC–MS/MS method. However, the limited number of reported cases is insufficient for making a precise assessment of false positive or negative rates. Research conducted using the UC Davis Veterinary Medical Teaching Hospital’s Small Animal Clinic database indicates that many cases are primarily diagnosed based on history, clinical signs, and human POC UDST. This preference for human POC UDST is likely because of its over-the-counter availability, rapid results, and affordability. It is estimated to be accurate in identifying barbiturates, opiates, benzodiazepines, and amphetamines/methamphetamines in dogs’ urine when these drugs are administered intravenously or orally. However, it is not as effective in identifying marijuana or methadone and has not been validated for phencyclidine or cocaine intoxication (29). In cases where POC UDST is used, the diagnosis is supported by a history and clinical examination findings consistent with drug exposure. Furthermore, LC–MS/MS test results require longer turnaround times, typically within 10–14 business days, and often do not alter the treatment plan or clinical course. However, it’s worth noting that the specificity and sensitivity of the POC UDST was conducted for only one kit and in a relatively small number of samples. POC UDSTs are qualitative and employ antibodies that may cross-react with structurally related compounds. For instance, a positive result for opiates may occur for all compounds in the same class, and there may be false positive results when some common medications or substances are used (as shown in Table 3, based on human data) (30, 31). It would be interesting to more thoroughly validate the commonly used POC UDSTs in dogs and cats.

**Table 3:** List of common medications that may cause false-positive results on urinary drug testing (30, 31).

Illicit drug	False-positive results due to cross-reactive medication/substance
Amphetamines	Amantadine, aripiprazole, atomoxetine, benzphetamine, bupropion, chlorpromazine, clobenzorex, doxepin, desipramine, dextroamphetamine, ephedrine, fenproporex, fluoxetine, isometheptene, labetalol, levomethamphetamine (active ingredient in some over-the-counter nasal decongestant inhalers), methylphenidate, phentermine, phenylephrine, phenylpropanolamine, promethazine, pseudoephedrine, ranitidine, selegiline, thioridazine, trazodone, trimethobenzamide, trimipramine
Barbiturate	Ibuprofen, naproxen
Benzodiazepines	Oxaprozin, sertraline
Cannabinoids	Dronabinol, efavirenz, hemp-containing foods, proton pump inhibitors, tolmetin and other nonsteroidal anti-inflammatory drugs
Cocaine	Coca-leaf tea, topical anesthetics containing cocaine
Opioids	Antibiotics (levofloxacin, ciprofloxacin), dextromethorphan, diphenhydramine, heroin, quetiqpine, quinine, quinolones, naloxone, poppy seeds, rifampin, tramadol, verapamil
Lysergic acid diethylamide (LSD)	Amitriptyline, chlorpromazine, diltiazem, doxepin, fentanyl, fluoxetine, metoclopramide, trazodone, bupropion, buspirone, risperidone, sertraline, verapamil, and methylphenidate
Phencyclidine	Dextromethorphan, diphenhydramine, doxylamine, ibuprofen, ketamine, lamotrigine, meperidine, thioridazine, tramadol, venlafaxine

At least one-third of the tests in this study were requested due to known or suspected cases of cruelty to animals. Violence is inherently linked to the culture of misuse of alcohol and drugs (32, 33). Unless these substances are used explicitly for medical purposes, any presence of illicit or recreational drugs in an animal specimen suggests an unintended exposure. Instances of malicious poisoning or various forms of animal abuse and cruelty, including involving pets in drug use, have been documented (34, 35). These are often exposures with the intent to harm. Finding these in animal specimens is a cause for concern and investigation. A pet owner who is aware that their animal may have ingested or been exposed to illegal substances may hesitate to acknowledge it (4, 8). Veterinarians should balance client confidentiality, best practice for the patients, and legal obligations.

It is important to remember that, given the large number of animal abuse or cruelty cases, the involvement of law enforcement, and the potential for criminal penalties, POC urine multidrug tests are merely screening tests (not confirmatory). Therefore, they might not be as useful to law enforcement. Samples should be sent to an established toxicological laboratory for the absolute confirmation of drugs of abuse (DA) in these cases.

This study has a few inherent limitations. First, the retrospective nature of the study, and the fact that it was based on information from referral forms or cases where the patient was found deceased, rather than comprehensive medical records, led to missing data. Moreover, cause-and-effect relationships often cannot be determined retrospectively. Second, the cohort size, although the largest yet, is nonetheless limited, which weakens the descriptive information and does not allow for statistical analyses. Third, the results of the DA screen test at CAHFS are qualitative rather than quantitative. For diagnostic purposes, this does not change the clinical course of the case since proof of exposure to these drugs is unequivocal. Nevertheless, it would have been interesting to compare drug concentrations to morbidity and mortality. Fourth, this study comprised clinical data from a single referral veterinary toxicological laboratory. Therefore, our results should be applied cautiously to other clinical settings.

In conclusion, in 75% of the drug-of-abuse screen tests conducted at CAHFS, the detected drug combinations included ATS. In 47% of cases, a combination of more than one drug group was found. In at least 32% of cases, the samples were submitted to CAHFS due to suspicion of cruelty against animals, and at least 41% of the patients were deceased when the samples were submitted. More studies on the prevalence of illicit drugs in small animals with confirmatory testing are warranted to fully understand the significance of this growing toxicological hazard in veterinary medicine.

## Data availability statement

The original contributions presented in the study are included in the article/supplementary material, further inquiries can be directed to the corresponding author.

## Ethics statement

Ethical approval was not required for the studies involving animals in accordance with the local legislation and institutional requirements because the work described in this manuscript involved the use of retrospective information from non-experimental (owned or unowned) animals. Established internationally recognized high standards (“best practice”) of veterinary diagnostics for the individual patient were always followed: ethical approval from a committee was therefore not specifically required for publication. No animals or people are identifiable within this publication, and therefore additional informed consent for publication was not required. Written informed consent was not obtained from the owners for the participation of their animals in this study because the work described in this manuscript involved the use of retrospective information from non-experimental (owned or unowned) animals. Established internationally recognized high standards (“best practice”) of veterinary diagnostics for the individual patient were always followed ethical approval from a committee was therefore not specifically required for publication. No animals or people are identifiable within this publication, and therefore additional informed consent for publication was not required.

## Author contributions

SK: Conceptualization, Data curation, Formal analysis, Investigation, Methodology, Project administration, Resources, Software, Supervision, Validation, Visualization, Writing – original draft, Writing – review & editing. CS: Conceptualization, Data curation, Formal analysis, Investigation, Methodology, Resources, Supervision, Validation, Visualization, Writing – review & editing. RP: Conceptualization, Data curation, Formal analysis, Funding acquisition, Investigation, Methodology, Project administration, Resources, Software, Supervision, Validation, Visualization, Writing – original draft, Writing – review & editing.
